# Alignment of India’s frontline One Health field epidemiology programme with the COHFE framework: a systematic curriculum mapping study

**DOI:** 10.3389/fpubh.2026.1910740

**Published:** 2026-08-17

**Authors:** Meera Dhuria, Adhiraj Mishra, Navneet Dhand, Hanul Thukral, Simmi Tiwari, Himanshu Chauhan, Priyanka Kundra, Bhavesh Lodhi Rajput, Kevisetuo A. Dzeyie, Ayanava Basu, Kamal Mushtofa, Aruna Sharma, Masaya Kato, Ranjan Das, Naveena B. Maheswarappa, Atul Goel

**Affiliations:** 1 National Centre for Disease Control, New Delhi, India; 2 Department of Animal Husbandry and Dairying, New Delhi, India; 3 World Health Organization Regional Office for South-East Asia, New Delhi, India; 4 Sydney School of Veterinary Science, The University of Sydney, Camden, NSW, Australia; 5 National Centre for Disease Control, SectorConnect Team, New Delhi, India; 6 World Health Organization – Country Office India, New Delhi, India

**Keywords:** capacity building, curriculum assessment, field epidemiology, India, One Health

## Abstract

**Background:**

The Competencies for One Health Field Epidemiology (COHFE) framework provides global standards for One Health field epidemiology training. India’s National Centre for Disease Control in collaboration with the Department of Animal Husbandry and Dairying developed the Field Epidemiology Programme in One Health (FEP OH), a three-month frontline training program. This study systematically assessed its curriculum alignment with the COHFE framework competencies to identify strengths and gaps.

**Methods:**

All 448 COHFE frontline competencies across 14 domains were systematically mapped against the FEP OH curriculum materials. Coverage was classified as covered, partially covered, or not covered through expert review and consensus validation involving program faculty and technical experts. Descriptive analyses examined coverage patterns by domain, sector-core category, and competency type.

**Results:**

Overall, 61.3% of competencies had at least some coverage (35.0% covered, 26.3% partially covered). COHFE One Health Core competencies showed strong alignment (82.8% coverage). Technical domains demonstrated variable coverage: data management and biostatistics (95.5%), field investigations (86.5%), surveillance systems (83.3%), and epidemiologic studies (78.8%) showed strong alignment, while ecosystem health (45.2%) and disease management (42.2%) had coverage gaps. Functional domains revealed marked disparities: communication (88.2%) had better coverage than training (20.0%) and ethics (17.1%). Sector-specific competencies showed lower alignment: public health (38.9%), animal health (35.7%), and environment (25.0%) than One Health competencies.

**Conclusion:**

This first global assessment of frontline One Health field epidemiology training against the COHFE framework demonstrates strong curriculum alignment of core multisectoral competencies but identifies critical coverage gaps in functional domains and sector-specific competencies. Findings provide an evidence-based roadmap for curriculum enhancement and offer a replicable methodology for other programs seeking to inform curriculum enhancement to support One Health workforce capacity.

## 1 Background

Emerging infectious diseases pose escalating threats to global health security, with more than 60% originating at the human-animal interface and over 70% of zoonotic pathogens originating from wildlife (1, 2). Based on India’s Integrated Disease Surveillance Program weekly outbreak reports data, between 2009–2013, a total of 191 probable and laboratory confirmed human zoonotic outbreaks were reported across India and that number nearly doubled to 388 during the next five years, i.e., from 2014–2018 (3)^1^. These events have exposed the inadequacy of siloed sectoral approaches, underscoring the essential role of the One Health approach in preventing, detecting, and responding to health threats at the human-animal-environment interface (4).

Field epidemiologists serve as frontline responders for disease surveillance, outbreak investigation, and emergency response, requiring competencies that span multiple sectors. Field Epidemiology Training Programs (FETPs), coordinated globally through networks such as the Training Programs in Epidemiology and Public Health Interventions Network, have been instrumental in building this workforce capacity worldwide (5–8). However, traditional FETPs have predominantly operated in sectoral silos, training either public health or veterinary epidemiologists separately, with minimal integration of environmental health expertise (9–12). This fragmented training approach has created workforce gaps in multisectoral collaboration, limiting capacity for effective preparedness and coordinated response to zoonotic and environmental health threats. One of the approaches to strengthening One Health field epidemiology capacity is curriculum development that explicitly integrates competencies across sectors. Competency frameworks for this purpose have recently emerged, studies systematically evaluating training curricula against such standards remain scarce, leaving programs without evidence on how well their content aligns with defined One Health competencies (13).

**Figure 1:**
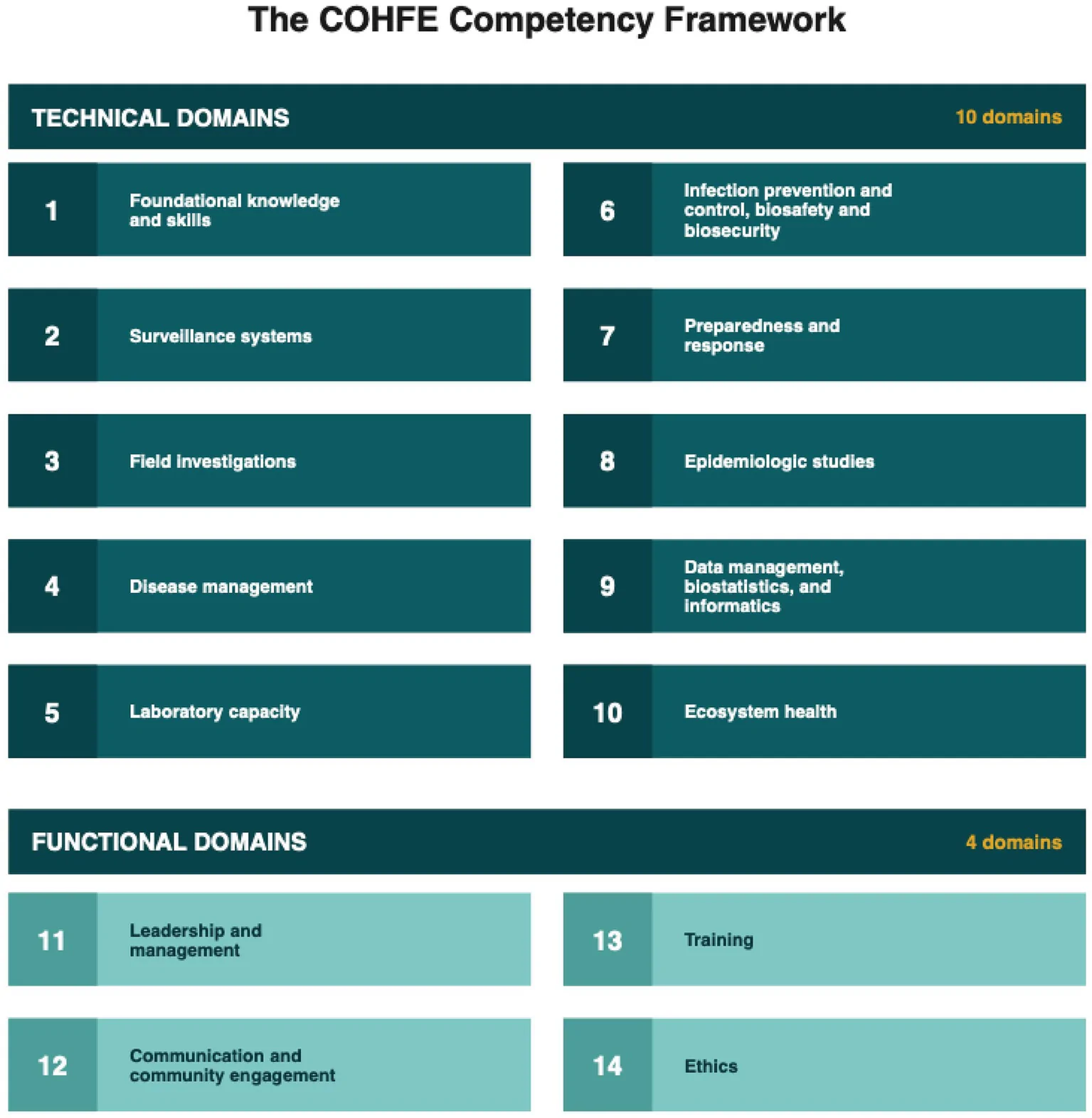
Structure of the competencies for one health field epidemiology (COHFE) framework, comprising 14 domains: 10 technical and 4 functional.

The Competencies for One Health Field Epidemiology (COHFE) framework, jointly developed by the Food and Agriculture Organization of the United Nations (FAO), the World Health Organization (WHO), and the World Organisation for Animal Health (WOAH), provides the first comprehensive global standard for One Health field epidemiology training (14). The framework encompasses 14 domains, including 10 technical domains (foundational knowledge and skills, surveillance systems, field investigations, disease management, laboratory capacity, infection prevention and control, preparedness and response, epidemiologic studies, data management and biostatistics, and ecosystem health) and 4 functional domains (leadership and management, communication and community engagement, training, and ethics) (Figure 1). This comprehensive framework defines competencies stratified across three levels: frontline (district-level), intermediate (state-level), and advanced (national/international-level). Competencies are further categorised as core One Health (essential for all programs), optional One Health (context-specific), and sector-specific (human, animal, or environment), enabling programs to adapt the framework to local needs while maintaining essential One Health integration. Across 14 domains, the framework specifies 448 frontline-level competencies (with additional competencies defined at intermediate and advanced levels). Each is a discrete statement classified as a knowledge, skill, or competency item; for example, a frontline surveillance competency reads ‘Analyse surveillance data using descriptive epidemiological and simple statistical methods’. The COHFE framework serves as both a curriculum design tool for existing programs and a blueprint for establishing new One Health field epidemiology training initiatives.

**Figure 2:**
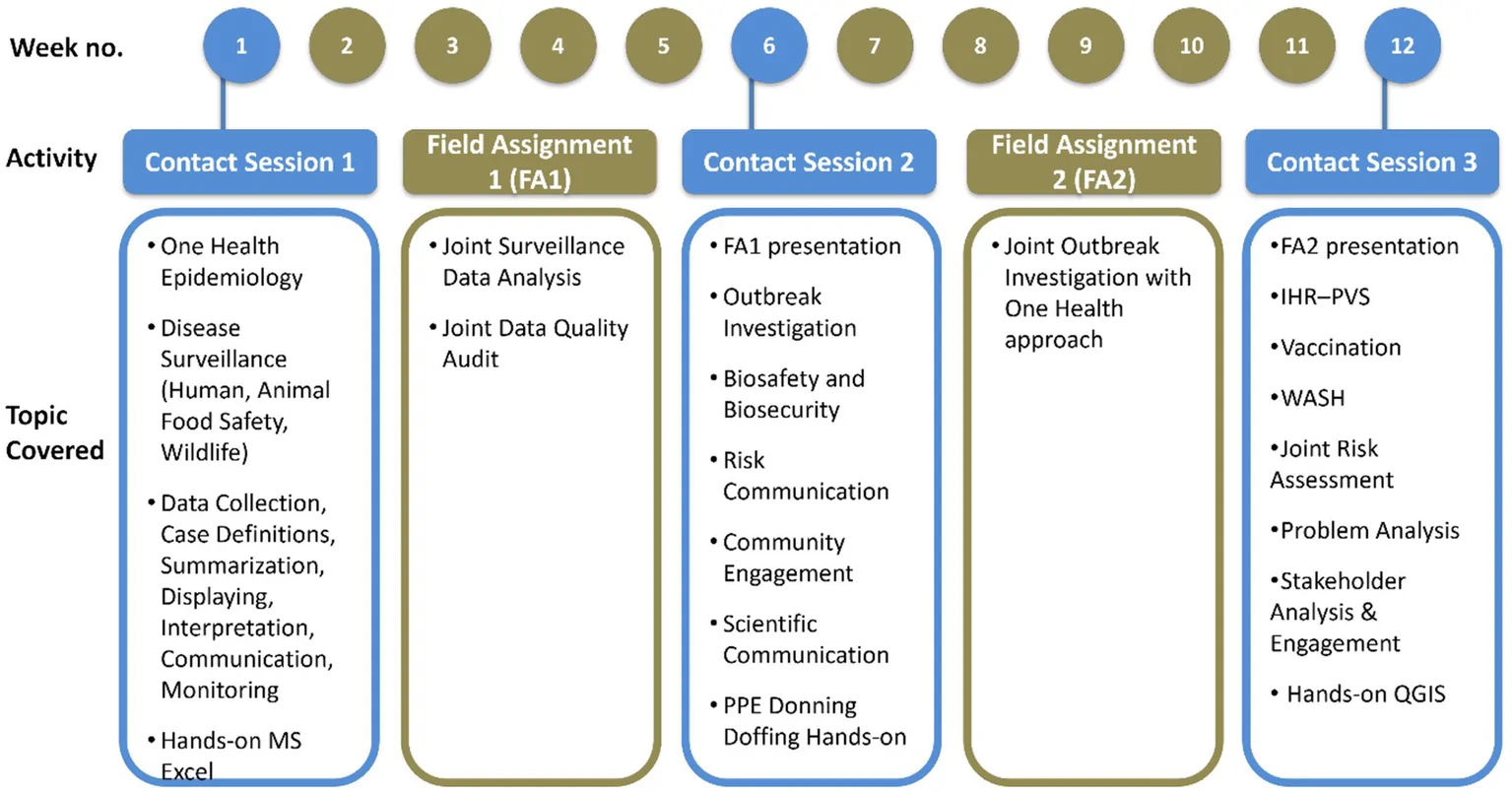
SectorConnect FEP OH programme structure and overview.

India’s National Centre for Disease Control (NCDC), in collaboration with the Department of Animal Husbandry and Dairying, developed the SectorConnect initiative to operationalise One Health principles in field epidemiology training. The SectorConnect Field Epidemiology Programme in One Health (FEP OH) is a 3-month on the job frontline-level training program that brings together district officers from public health, animal husbandry, wildlife, food safety, international health, and other relevant sectors. Following the successful 3×3 Frontline FETP model in India that trained over 300 public health officers across 124 districts in eight states between Jan 2021 and May 2023 (15), the FEP OH program integrated multisectoral competencies through a competency-based learning approach combining classroom instruction (approximately 10% of time) and mentored field assignments (approximately 90% of time) (16)^2^. The training’s design and curriculum were developed through a series of national level multisectoral stakeholder consultations, involving stakeholders from public health, animal health, wildlife health and environment health sectors at international, national and state levels (Figure 2 and Supplementary Table 1). The core competencies of the program include (i) Epidemiological concepts and skills (ii) Surveillance data analysis, interpretation and sharing, (iii) Joint outbreak investigation using One Health approach, (iv) Effective risk communication and community engagement, (v) Scientific communication and (vi) Epidemiological software competencies. Since its launch in 2023, the program has trained 473 officers representing public health, animal husbandry, food safety and wildlife health sectors across 6 states of India. SectorConnect’s secretariat is anchored at the NCDC, Delhi, which implements the program in collaboration with states and relevant stakeholders, resulting in institutionalisation of the program by the country. The program jointly trains multisectoral field officers responsible for investigating and responding to public and animal health emergencies as members of district rapid response teams.

While the FEP OH program was designed to incorporate One Health principles, no systematic assessment of its alignment with the COHFE framework (or, to our knowledge, of any frontline One Health field epidemiology curriculum against the COHFE framework) has been conducted. This represents a critical gap: without competency mapping, coverage strengths and gaps remain unidentified, and programs lack an evidence base for curriculum refinement Curriculum alignment analysis is essential for identifying coverage gaps, ensuring comprehensive competency development, and informing evidence-based curriculum refinement. Such mapping exercises provide critical insights not only for program improvement but also for other countries seeking to develop or adapt One Health field epidemiology training programs. Understanding the extent and pattern of COHFE framework coverage in operational training programs can identify design strengths and coverage gaps and inform practical guidance for translating global competency frameworks into context-specific curricula. However, as this addresses the intended curriculum rather than its delivery, it does not capture implementation challenges, which require study of how the curriculum is taught and learned.

Strengthening field epidemiology training capacity aligns with multiple global health security initiatives. The Quadripartite One Health Joint Plan of Action (2022–2028), jointly developed by FAO, the United Nations Environment Programme, WHO, and WOAH, prioritises the development of sustainable One Health capacities within health systems (4). Such training programs directly support the implementation of the International Health Regulations (IHR 2005) by enhancing countries’ capacities to detect, assess, notify, and respond to public health events, and complement the WOAH’s Performance of Veterinary Services (PVS) Pathway by strengthening veterinary workforce competencies (17). One Health field epidemiology training programs are in the early stages of development, with the first global reference framework launched in 2024, and an increasing number of countries subsequently adopting and implementing such programs (18–21). Systematic evaluation of training program alignment with global frameworks such as the COHFE framework provides evidence for monitoring progress toward these international commitments.

This study aimed to systematically map the NCDC SectorConnect FEP OH curriculum against the COHFE framework frontline competencies to assess alignment and identify coverage gaps. Specific objectives were to: (i) determine the coverage of COHFE domains and subdomains in the FEP OH curriculum across knowledge, skills, and competency levels; (ii) analyse coverage patterns across One Health core, One Health optional, and sector-specific (human health, animal health, and environment) competencies; and (iii) identify priority areas for curriculum enhancement to strengthen One Health integration and comprehensive competency development.

## 2 Methods

### 2.1 Study design and framework

This was a curriculum-mapping study to assess the alignment of the NCDC SectorConnect curriculum with the COHFE framework as the reference standard. Curriculum mapping is an established approach in education research for assessing alignment between intended curricula and reference standards (22). This analysis focused exclusively on frontline-level competencies, which correspond to the district-level training objectives of the SectorConnect program. Competency type was classified per the COHFE framework as knowledge (K), skill (S), or competency (C), where knowledge refers to factual/theoretical assimilation, skill to the applied ability to complete tasks, and competency to the proven ability to integrate knowledge and skills in practice. See definitions and explanation in the COHFE framework (11).

### 2.2 Study setting and program description

The SectorConnect is a 3-month (12-week) competency-based field epidemiology training program for district-level officers from multiple sectors, including public health, veterinary services, wildlife, food safety, international health, and other relevant departments. The program follows an experiential learning model with approximately 10% of training time allocated to classroom contact sessions and 90% to mentored field assignments. Training content is delivered through 3-day contact sessions interspersed with two field assignment periods. During contact sessions, participants receive instruction on specific epidemiological concepts and skills, which are then applied during field intervals through practical assignments under faculty mentorship. This study analysed curriculum materials from the implementation of the program.

### 2.3 Data sources

Two primary data sources were utilised for this analysis. First, the COHFE framework document provided a complete set of frontline-level competencies across all 14 domains (10 technical and 4 functional). Second, SectorConnect curriculum materials included the course outline, module descriptions, learning objectives for each contact session, session plans, training presentations, and field assignment guidelines. These materials comprehensively documented the intended curriculum content delivered during the three contact sessions and two field assignment periods.

Frontline-level competencies were available from the COHFE framework, organised in a structured Excel database. Each competency entry was characterized by multiple attributes: domain number and name (1–13, 20), subdomain number and name, sector designation (One Health, Public Health, Animal Health, or Environment), core status (core or optional), sector-core category (One Health Core, One Health Optional, Public Health, Animal Health, or Environment), competency level (Frontline), unique competency identifier (key), competency statement text, and competency type (Knowledge, Skill, or Competency). This structured organisation enabled systematic comparison with curriculum content.

### 2.4 Curriculum mapping process

The curriculum mapping was conducted through a multi-phase process involving expert review and consensus building. Initially, the SectorConnect curriculum content was mapped against COHFE competencies for the first seven domains (Domains 1–7) by a single reviewer who is not a member of the SectorConnect faculty and is not involved in program implementation, providing an independent review of these domains. This process documented which competencies were addressed within specific modules, learning objectives, and field assignments. A training session was then conducted to explain the mapping approach and classification criteria to the broader team. Each COHFE domain was later assigned to two panel members: one from the program implementers (NCDC or DAHD) and one from WHO, who reached consensus on the mapping of each KSC statement, so that no domain was mapped by program implementers alone. Subsequently, a one-day workshop was organised involving the NCDC program faculty and the WHO South-East Asia Region technical staff to review the initial mappings. During this workshop, participants reviewed each mapped competency and classified whether they agreed with the assessment. When disagreement occurred, team members provided justification for alternative classifications. This workshop served both as a quality assurance mechanism and as a capacity-building exercise for the mapping methodology. During the workshop, the NCDC and WHO team collaboratively mapped competencies in the remaining seven domains (Domains 8–14) using the same systematic approach. Any ambiguities or classification challenges were discussed among team members at another workshop and via email until consensus was reached. Each mapping was anchored to a specific reference from the SectorConnect learning modules, and any change to a statement’s mapping or classification required a documented justification.

### 2.5 Coverage classification system

Each COHFE competency was classified into one of three mutually exclusive categories based on its representation in the FEP OH curriculum. Competencies were classified as “Covered” when they were explicitly and adequately addressed in curriculum materials, with specific modules, learning objectives, or field assignments targeting that competency. Competencies were classified as “Partially covered” when they were addressed in the curriculum but with limited depth, missing key components, or requiring additional content to achieve full competency development. Competencies were classified as “Not covered” when they were not addressed in any curriculum materials, modules, learning objectives, or field assignments. Classification decisions were based on expert judgment by team members with expertise in field epidemiology, One Health, and curriculum development. For each competency, mappers documented the specific SectorConnect module, learning objective, or assignment that addressed the competency, along with justification for the coverage classification.

### 2.6 Data management and standardisation

All mapping data were recorded in Microsoft Excel spreadsheets, with a separate sheet for each domain. After mapping, data from all 14 domain sheets were consolidated into a single long-form dataset. A standardisation process was implemented to ensure consistency in coverage classifications across domains and reviewers. This included standardising text entries for coverage categories (e.g., “fully covered,” “Fully covered,” and “Full covered” were all standardised to “Covered”), trimming extraneous spaces, and resolving case inconsistencies. Quality assurance checks identified competencies with coverage classifications but missing module or justification information, which were subsequently reviewed and corrected.

### 2.7 Data analysis

Descriptive statistical analyses were conducted using R version 4.3.3 with RStudio. For each competency, the final coverage classification was determined by prioritising updated classifications from the workshop review process when available, otherwise retaining the initial classification. Coverage statistics were calculated across multiple dimensions. Domain-level analysis examined coverage distribution across all 14 COHFE domains. Sector-core analysis assessed coverage patterns for One Health Core competencies (essential for all programs), One Health Optional competencies (context-dependent), and sector-specific competencies (Public Health, Animal Health, and Environment). Competency type analysis evaluated coverage across Knowledge, Skill, and Competency categories.

For all analyses, percentage alignment was calculated as the proportion of competencies that were either covered or partially covered: (covered + partially covered) / total * 100. Results were summarised in frequency tables and cross-tabulations. Summary statistics and graphs were generated to identify domains, sectors, and competency types with minimal to strong alignment, thereby highlighting curriculum strengths and coverage gaps. For standardized interpretation of the analysis, SectorConnect’s alignment with the COHFE framework was graded into four equal-width categories: Minimal alignment (0–24%), Limited alignment (25–49%), Moderate alignment (50–74%), and Strong alignment (75–100%).

### 2.8 Ethical considerations

This study involved an analysis of curriculum documents and competency frameworks and did not involve human subject research. No ethical approval was required for this document analysis study.

## 3 Results

### 3.1 Overall competency coverage

A total of 448 frontline-level competencies from the COHFE framework were analysed for coverage in the SectorConnect curriculum. Overall, approximately 60% of competencies had at least some level of coverage in the curriculum, with the remaining competencies not addressed (Table 1).

**Table 1:** Overall coverage of the COHFE frontline competencies in the field epidemiology programme in one health (FEP OH) curriculum in India.

Category	Number (n)	Percentage (%)
Total competencies	448	100
Covered	157	35
Partially covered	118	26.3
Not covered	173	38.6

### 3.2 Alignment by the COHFE domain

**Figure 3:**
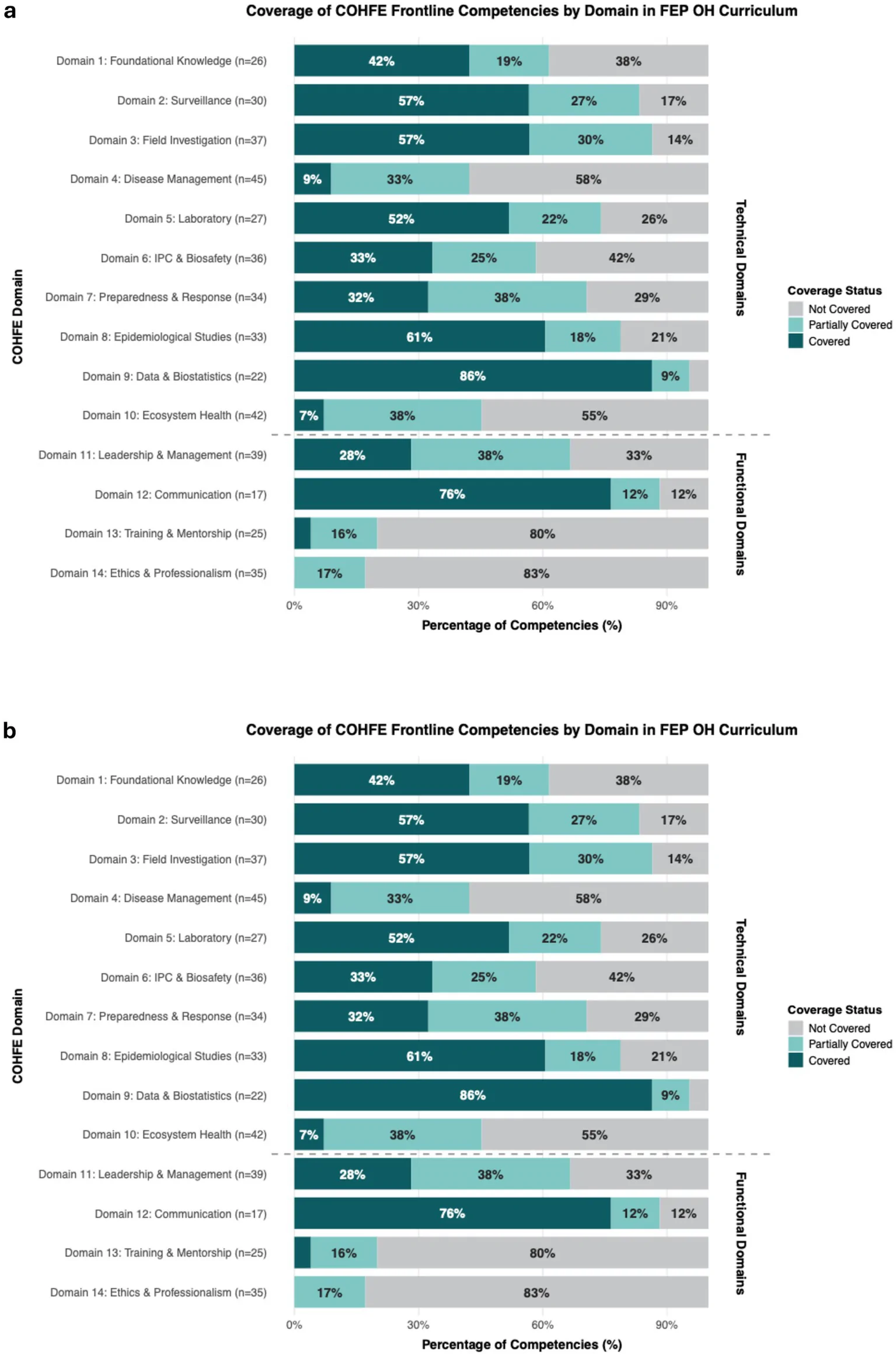
Distribution of coverage status (covered, partially covered, not covered) across COHFE competency domains in the field epidemiology programme in one health (FEP OH) curriculum in India: **(a)** Overall and **(b)** Only for one health core competencies.

Alignment varied substantially across the 14 COHFE domains, ranging from less than 20% to over 95% (Figures 3a,b). Among technical domains, data management and biostatistics (Domain 9) demonstrated the strongest alignment, with >95% competencies covered. Core epidemiological functions, including surveillance systems (Domain 2), field investigations (Domain 3), and epidemiologic studies (Domain 8), also showed strong alignment with the COHFE framework.

Laboratory capacity (Domain 5) and preparedness and response (Domain 7) demonstrated moderate alignment of >70%. In contrast, some technical domains showed substantial coverage gaps. Ecosystem health (Domain 10) and disease management (Domain 4) had less than half of their competencies covered showing limited alignment. Foundational knowledge and skills (Domain 1) and infection prevention and control (Domain 6) showed moderate alignment with the COHFE framework at >50%.

Among functional domains, communication and community engagement (Domain 12) was well represented in the curriculum, approaching alignment levels similar to those of the strongest technical domains. Leadership and management (Domain 11) showed moderate alignment at >60%. However, training (Domain 13) and ethics (Domain 14) competencies were minimally addressed, with less than one-quarter of competencies in these domains having any coverage. The granular numbers for the coverage by domain is provided in Supplementary Table 2.

### 3.3 Alignment by sector-core category

Analysis by sector-core designation revealed a clear hierarchy in competency coverage (Table 2). One Health Core competencies, designated as essential for all field epidemiology programs, demonstrated strong alignment with over 80% covered or partially covered. One Health Optional competencies showed moderate alignment at approximately 60%.

**Table 2:** Coverage of COHFE frontline competencies by sector in the field epidemiology programme in one health (FEP OH) curriculum in India.

Sector category	Total	Covered	Partially covered	Not covered	Total coverage (%)
One health (core)	151	87	38	26	82.8
One health (optional)	195	59	57	79	59.5
Public health	18	4	3	11	38.9
Animal health	56	6	14	36	35.7
Environment	28	1	6	21	25.0

Sector-specific competencies showed limited alignment across all three sectors. Public Health, Animal Health, and Environment sector-specific competencies all had less than 40% coverage, with Environment showing the lowest representation at one-quarter coverage. This pattern indicates that while the program addressed core multisectoral One Health competencies well, integration of sector-specific optional competencies, particularly in animal and environmental health, was limited.

### 3.4 Alignment by competency type

Coverage analysis by competency classification revealed relatively uniform distribution across knowledge, skills, and competency levels (Table 3). All three types showed approximately 60% alignment, with no substantial differences between them.

**Table 3:** Coverage of COHFE frontline competencies by competency type in the field epidemiology programme in one health (FEP OH) curriculum in India.

competency type	Total	Covered	Partially covered	Not covered	Alignment/coverage (%)
Competency	132	42	40	50	62.1
Skill	171	66	40	65	62.0
Knowledge	145	49	38	58	60.0

### 3.5 Quality assurance and consensus review

Of the 448 competencies analysed, 235 competencies from the first seven domains were reviewed during the one-day consensus workshop. The review panel endorsed the single reviewer’s initial classification for 203 competencies (86.4%) and revised 32 competencies (13.6%) following structured discussion and documented justification. As the initial mapping was performed by a single reviewer, this figure reflects the panel’s concordance with the initial classifications rather than a formal inter-rater agreement statistic. The remaining 213 competencies from Domains 8–14 were collaboratively mapped by the NCDC and WHO team using the established methodology.

## 4 Discussion

To our knowledge, this is the first formal systematic mapping of a frontline One Health field epidemiology training curriculum against the COHFE framework globally. This study assessed the alignment of India’s SectorConnect with the 448 frontline competencies defined in the COHFE framework. The analysis revealed overall alignment of 61.3%, with substantial variation across domains and competency categories. The program demonstrated strong alignment of One Health Core competencies (82.8%) and technical epidemiological domains, including data management, surveillance, field investigations, and epidemiologic studies (all exceeding 75% coverage). In contrast, marked coverage gaps were evident in functional domains, particularly training (20%) and ethics (17%), and in sector-specific competencies, especially environment (25%) and animal health (36%). These findings provide an evidence-based foundation for curriculum enhancement and offer insights for other countries developing or evaluating One Health field epidemiology training programs.

### 4.1 Strengths of the SectorConnect curriculum

The SectorConnect curriculum demonstrated several notable strengths in operationalising the One Health approach at the frontline level. The strong alignment of One Health Core competencies (those designated as essential for all field epidemiology programs regardless of context) indicates that the program successfully prioritises fundamental multisectoral competencies. The near-complete coverage of data management and biostatistics competencies, combined with strong representation of surveillance systems and field investigation skills, reflects appropriate emphasis on the core technical functions that frontline epidemiologists perform daily. Also, the strong alignment of communication and community engagement competencies is particularly valuable, as these skills are critical for outbreak response, risk communication, and community mobilisation at the district level. The balanced alignment across knowledge, skills, and competencies suggests the curriculum addresses not only theoretical understanding but also practical application and integration of competencies. This curriculum foundation is intended to support graduates in functioning within multisectoral teams and respond to routine and emergency public and animal health events.

### 4.2 Coverage gaps in functional domains

The minimal alignment of training (Domain 13) and ethics (Domain 14) competencies represents a significant gap with practical implications in relation to the COHFE framework. Frontline field epidemiologists frequently encounter situations requiring these competencies: training community health workers and junior staff, mentoring colleagues from other sectors, navigating consent and confidentiality issues during outbreak investigations, and making decisions about resource allocation under uncertainty. The low coverage may reflect programmatic priorities that emphasise technical over functional competencies, time constraints within the three-month format, or assumptions that these competencies are better addressed at intermediate or advanced levels. However, ethical dilemmas and training responsibilities often arise at the frontline, and graduates lacking these competencies may be inadequately prepared for real-world challenges. The COHFE framework explicitly includes these domains at the frontline level, recognising their importance for district-level practice (13). Strengthening these areas could involve integrating ethical case discussions throughout existing modules and incorporating training skills development into field assignment supervision and presentation activities.

### 4.3 Coverage gaps in sector-specific and selected technical competencies

The limited alignment of sector-specific competencies for animal health and environment contrasts sharply with the strong alignment of One Health Core competencies, revealing a tension between breadth and depth in multisectoral training. It is important to note that sector-specific competencies in the COHFE framework are designated as optional, allowing programs to tailor content to local needs and available expertise. In addition, certain parallel training programs specific to sectoral needs like Public Health Emergency and Disaster Management Professional Development Programme, Noncommunicable Disease Field Epidemiology Training Program, certificate courses on wildlife management, control of illegal wildlife trade and nature conservation are running in the country to fill that gap (23–25, 28)^3^. Nevertheless, the marked gap in environmental health competencies reflects a challenge encountered globally in One Health training initiatives. Environmental and ecosystem health integration remains nascent in most field epidemiology programs, with limited faculty expertise, fewer established curricula, and less institutional infrastructure compared to human and animal health sectors.

This broader environmental challenge is reflected in the limited alignment of Domain 10 (Ecosystem health). This domain encompasses competencies in environmental determinants of health, land use patterns, biodiversity monitoring, and climate change impacts, increasingly recognized as relevant to disease emergence and One Health surveillance. At the district level, the appropriate target for the frontline level is not specialist expertise in these areas but the ability to recognise environmental signals relevant to disease emergence and to engage the appropriate wildlife, environmental, or veterinary counterparts, emphasising awareness, basic assessment, and referral rather than in-depth technical mastery. The FAO has recently developed the Field Training Programme for Wildlife, Ecosystems, Biodiversity, and the Environment (FTP-WEBE) to address this gap by building capacity specifically in environmental surveillance and wildlife health (26)^4^. While FTP-WEBE represents an important advancement, substantial work remains to effectively integrate environmental competencies into multisectoral One Health programs like SectorConnect. For the SectorConnect program, strategic partnerships with environmental and wildlife institutions, targeted faculty development, and adaptation of FTP-WEBE materials could strengthen this critical component. The challenge is particularly relevant for a country like India with its rich biodiversity, extensive human-wildlife interface, and emerging zoonotic disease risks from wildlife reservoirs.

Domain 4 (Disease management) coverage showed unexpectedly low coverage given its direct relevance to outbreak response. This domain includes competencies in clinical aspects of infectious diseases, vaccination strategies, treatment protocols, antimicrobial resistance, and disease control interventions. The gap may indicate that the program emphasises epidemiological investigation over clinical management, potentially reflecting the composition of training cohorts and the primary focus on surveillance rather than treatment. However, frontline epidemiologists often must understand disease management principles to effectively investigate outbreaks, interpret clinical case definitions, and coordinate with healthcare providers. Strengthening this domain could involve closer collaboration with clinical faculty and incorporating case management considerations into outbreak investigation modules.

### 4.4 Implications for program structure and the COHFE framework

A critical finding from this mapping exercise concerns the practical challenge of incorporating all COHFE frontline competencies into a three-month training program. The SectorConnect program faculty reported difficulty fitting comprehensive content across 14 domains into the available time while maintaining the essential balance between classroom instruction (10%) and field application (90%). This challenge highlights an important gap in the COHFE framework implementation guidance: while the framework comprehensively defines the competencies field epidemiologists should possess, and includes supplementary manuals on curriculum development, mentorship, and evaluation, there remains limited practical guidance on how to integrate and deliver competencies efficiently within time-constrained programs.

The COHFE framework is accompanied by guidance on prioritising optional competencies to suit country contexts, and it clearly delineates competencies across frontline, intermediate, and advanced levels. However, what is needed is a practical illustration of how the numerous competencies can be woven together across modules rather than addressed domain by domain. We recommend that the Quadripartite organisations consider developing illustrative curriculum models or case examples demonstrating successful integration strategies. They could also show how a single training module or field assignment can address competencies from multiple domains simultaneously, how competencies naturally cluster around common epidemiological activities, and how experiential learning through field assignments can efficiently develop multiple competencies in context. This practical implementation guidance would enhance the framework’s utility for program developers worldwide who are working within real resource constraints while striving for comprehensive competency development. It should be noted that alignment with the COHFE framework reflects coverage of a consensus-based competency framework and should not be interpreted as evidence of training effectiveness. The relationship between COHFE competency coverage and field performance has not yet been empirically established.

### 4.5 Contextual considerations for India and the global south

The SectorConnect program operates within the context of India’s decentralised health system and resource constraints typical of many countries in the Global South. District-level officers often work with limited laboratory infrastructure, variable surveillance systems, competing priorities, and large populations. The program’s strong emphasis on foundational epidemiological skills (surveillance, outbreak investigation, data analysis) appropriately reflects these realities and the immediate needs of frontline workers. The multisectoral district team model, bringing together officers from human health, veterinary, wildlife, and other sectors, represents an innovative approach to fostering collaboration despite organisational silos and limited resources.

For countries in the Global South, the tension between comprehensive competency coverage and practical feasibility is particularly acute. Faculty expertise may be concentrated in certain sectors (typically human health), resources for extensive training programs may be limited, and participants may be unable to be released from their duties for extended periods. The SectorConnect ‘s three-month duration with 90% field-based learning reflects these pragmatic considerations while maintaining competency development. This model of shorter, intensive, practice-based training may be more sustainable and scalable in resource-limited settings than longer residential programs. However, it also necessitates careful prioritisation of competencies and may require complementary mechanisms such as continuing education, advanced training for selected individuals, and just-in-time learning resources to address competencies not fully covered in the foundational program.

### 4.6 Methodological considerations and limitations

This study employed a rigorous systematic approach to curriculum mapping, including independent initial assessment, consensus-based validation through a collaborative workshop, and standardised data management. The involvement of both program implementers and technical experts in the mapping process enhanced validity.

However, several limitations warrant consideration. First, the analysis assessed curriculum content rather than actual implementation or learner competency achievement. Competencies classified as “covered” in curriculum documents may not be adequately taught, learned, or retained. Second, the classification of coverage levels involved expert judgment with inherent subjectivity, despite efforts to achieve consensus. Different reviewers might assess the adequacy of coverage differently, particularly for the “partially covered” category. Relatedly, our design did not generate the independent, parallel classifications of the same competencies by two or more raters that are required to compute inter-rater reliability coefficients such as Cohen’s or Fleiss kappa. The initial mapping of Domains 1–7 was performed by a single reviewer and subsequently endorsed or revised by a review panel, while Domains 8–14 were mapped collaboratively. We therefore report the panel’s concordance with the initial mapping as a quality-assurance measure rather than a formal reliability coefficient. Future curriculum-mapping work, including the planned assessment of the intermediate Fellowship curriculum, should incorporate independent duplicate coding by multiple reviewers with formal kappa reporting. Third, this analysis focused solely on the frontline program; the intermediate Fellowship program was not assessed, and some competencies may be more appropriately addressed at that level. Fourth, our alignment metric combines covered and partially covered competencies, weighting the two equally; readers should therefore interpret it alongside the separately reported full-coverage figures. Finally, the findings represent a single program in one country context and may not be generalisable to other settings with different needs, resources, and priorities. However, the systematic and transparent methodology employed provides a replicable model for other programs to conduct similar assessments and offers valuable baseline data for evidence-based curriculum enhancement and ongoing quality assurance.

### 4.7 Recommendations for curriculum enhancement

Based on the identified coverage gaps, we recommend several strategies for strengthening the SectorConnect curriculum. For training and ethics competencies, rather than adding standalone modules, we suggest integrating these competencies throughout existing content. Ethical case studies can be incorporated into outbreak investigation modules; training skills can be developed through peer-teaching exercises during contact sessions; and field assignments can include mentoring or teaching components. For ecosystem health and environmental competencies, targeted partnerships with environmental institutions, wildlife departments, and research organisations could provide specialised expertise. Adapting materials from the FAO FTP-WEBE curriculum and engaging environmental faculty for specific sessions could enhance this content without requiring comprehensive expertise from all program faculty.

For animal health competencies, deeper engagement with the Department of Animal Husbandry and Dairying could strengthen sector-specific content. Inviting veterinary epidemiologists as guest faculty, conducting joint field visits to animal health facilities, and incorporating more veterinary case studies from other programs, such as Asia Pacific Consortium of Veterinary Epidemiology (12), would enhance integration of animal health. Some of this is already happening, but further work may be required to strengthen collaborations. For disease management competencies, collaboration with infectious disease clinicians and inclusion of clinical case management considerations in outbreak investigation training would address this gap, though key concepts of public, animal and environmental health management are covered in sector-specific training programmes running in parallel in the country (23–25).

Given time constraints, prioritisation is essential. The program might focus on ensuring all One Health Core competencies are fully addressed while selectively strengthening high-priority optional and sector-specific competencies based on India’s disease burden and program objectives.

### 4.8 Contributions to global health security

This evaluation contributes to strengthening field epidemiology capacity in alignment with multiple global health security frameworks. The Quadripartite One Health Joint Plan of Action (2022–2028) prioritises building sustainable multisectoral capacities for health systems. Systematic alignment with the COHFE framework directly supports this priority by ensuring training programs develop the competencies needed for effective One Health implementation. The SectorConnect program contributes to India’s implementation of the International Health Regulations (2005) by strengthening capacity to detect, assess, notify, and respond to public health events, particularly those at the human-animal-environment interface, while also addressing applied epidemiology knowledge and skill gaps in veterinary human resources, highlighted in the PVS India report (27)^5^. As countries scale One Health field epidemiology training, systematic curriculum evaluation against global standards provides essential evidence for monitoring progress toward these international commitments and ensuring quality and consistency across programs.

## 5 Conclusion

This study provides the first systematic evaluation of a frontline One Health field epidemiology training curriculum against the COHFE framework. The NCDC SectorConnect program demonstrates strong alignment with One Health Core competencies and foundational epidemiological functions, establishing a solid foundation for multisectoral collaboration at the district level. However, significant coverage gaps exist in functional domains (training and ethics), sector-specific competencies (particularly in animal health and the environment), and selected technical areas, offering clear opportunities for curriculum enhancement.

The findings highlight both the promise and the challenges of translating comprehensive competency frameworks into practical, time-constrained training programs in resource-limited settings. Addressing identified coverage gaps through strategic curriculum revision, faculty development, and institutional partnerships will strengthen the program’s ability to develop the multisectoral workforce needed to address emerging health threats at the human-animal-environment interface.

As countries worldwide scale up One Health capacity in response to pandemic preparedness commitments and global health security imperatives, systematic alignment with the COHFE framework provides essential guidance for workforce development. This evaluation demonstrates that rigorous curriculum assessment can inform evidence-based program improvement, ensuring that field epidemiology training effectively aims to prepare frontline professionals to detect, investigate, and respond to health threats in an increasingly interconnected world. Continued investment in One Health field epidemiology training, combined with ongoing evaluation and adaptation, will strengthen India’s health security infrastructure and contribute to regional and global preparedness for future health emergencies.

## Data Availability

The raw data supporting the conclusions of this article will be made available by the authors, without undue reservation.

## Footnotes

1
https://ncdc.mohfw.gov.in/includes/SideBar/ncdc_newsletter.php

2
https://www.tephinet.org/sectorconnect-enhancing-preparedness-integrated-response-india

3
https://wii.gov.in/certificate-course-in-wildlife-management

4
https://www.fao.org/animal-health/news-events/news/detail/fao-enhances-one-health-capacity-in-wildlife-and-environment-sectors/en

5
https://www.woah.org/app/uploads/2021/03/25022019-india-pvs-evaluation-report-final.pdf
