# Supplementary material for: Alignment of India’s frontline One Health field epidemiology programme with the COHFE framework: a systematic curriculum mapping study
Source: Front Public Health. 2026 Aug 17;14:1910740. doi: 10.3389/fpubh.2026.1910740 (PMC13527032; doi:10.3389/fpubh.2026.1910740)
Supplement: Supplementary file 1 [file Table_1.docx]

**Supplementary File 1: List of the member institution in SectorConnect Alliance**

- All India Institute of Hygiene and Public Health, Kolkata, West Bengal
- All India Institute of Medical Sciences, Bibinagar, Telangana
- Central Zoo Authority, New Delhi
- Food and Agriculture Organization of the United Nations – India office
- Food Safety and Standards Authority of India, New Delhi
- Guru Angad Dev Veterinary and Animal Sciences University, Ludhiana, Punjab
- Indian Council of Agricultural Research (ICAR)- National Institute of Veterinary Epidemiology and Disease Informatics, Bengaluru, Karnataka
- ICAR-National Institute of Foot and Mouth Disease, Bhubaneswar, Odisha, India
- ICAR-National Institute of High Security Animal Diseases, Bhopal, Madhya Pradesh
- Indian Council of Medical Research (ICMR)-National Institute of Epidemiology, Chennai, Tamil Nadu
- ICAR-Indian Veterinary Research Institute, Bareilly, Uttar Pradesh
- ICMR, New Delhi
- Institute of Animal Health and Veterinary Biologicals, Bengaluru, Karnataka
- Karnataka Veterinary, Animal and Fisheries Sciences University, Bidar, Karnataka
- Kerala Veterinary and Animal Sciences University, Pookode, Kerala
- National Health Systems Resource Centre, New Delhi
- National Institute of Health & Family Welfare, New Delhi
- National Institute of Public Health Training & Research, Mumbai, Maharashtra
- North-Eastern Indira Gandhi Regional Institute of Health and Medical Sciences, Shillong, Meghalaya
- Regional Institute of Medical Sciences, Imphal, Manipur
- South Asia Field Epidemiology and Technology Network, India office
- United States Centers for Disease Control and Prevention - India office
- Wildlife Institute of India, Dehradun, Uttarakhand
- World Health Organization – India office
- World Organization for Animal Health, Paris
- Zoological Survey of India, Kolkata, West Bengal

**Supplementary Table 1. Modules and respective learning objectives of the SectorConnect Field Epidemiology Programme in One Health (FEP OH), India**

| **Module no.** | **Module Name** | **Learning Objectives** |
| --- | --- | --- |
| 1.01 | Introduction to One Health | - Discuss what is One Health - Describe One Health Approach - Foundation of One Health - List down common One Health Issues - Examples of successful One Health approaches |
| 1.02 | Introduction to Epidemiology | - Define epidemiology and describe what each of the components of the definition mean - Summarize the epidemiologic approach of a field epidemiologist - Describe the application of epidemiology to the range of public health problems |
| 1.03 | Disease Surveillance | - Understand One Health and its importance in the 6 steps of the disease surveillance cycle - Disease Reporting Systems in Human Health, Animal Health, food Safety and Wildlife Health. - Identify and describe the flow of surveillance data in your area - Summarize the purpose and use of local surveillance data |
| 1.04 | Collecting Surveillance Data | - Identify the reportable diseases or conditions for your district - Explain the difference between passive and active data collection - Describe basic data collection methods - Summarize the rationale for zero reporting - Describe limitations of reporting systems and ways to improve reporting - Describe why it is important to use a One Health approach to share data and information across relevant sectors |
| 1.05 | Case Definitions and Line Lists | - Describe a case definition - Explain why use of a consistent case definition is important for surveillance - Determine if a patient or animal meets a case definition - Enter data into a line list - Apply a One Health approach to case definitions |
| 1.06 | Understanding  Data Quality | - Name and identify data quality issues that can affect disease surveillance reporting - Describe some consequences of poor data quality - Explain the steps needed to promote good data quality |
| 1.07 | Summarising Data | - Explain the difference between quantitative and qualitative variables and how to summarize each - Explain and calculate:   - measures of central location: mean, median and mode   - measures of spread: range   - measures of disease frequency: ratio, proportion, rate, prevalence, incidence, attack rates, mortality rates, case-fatality rate |
| 1.08 | Displaying Data | - Explain the value of organizing and displaying data - Describe the methods for organizing and displaying data - Select a table, graph, or map that is appropriate for the data - Use paper and pencil to create tables, graphs, and maps - Incorporate One Health concepts into organizing and displaying data |
| 1.09 | Hands on MS Excel | - Open a workbook, create and save a worksheet - Enter, edit, and format data in a database - Use formulas and functions to summarize and analyse data - Organize data by sorting and filtering - Chart data using a histogram and line graph - Use Excel to perform district data surveillance functions — enter, manage, and analyse data |
| 1.10 | Interpretation of Data | - Describe and interpret summarized data - Describe the use of thresholds when analysing surveillance data - List possible reasons for an observed increase in reported cases - Incorporate One Health concepts into the interpretation of human, animal, and environmental data |
| 1.11 | Communicating Surveillance Information | - Explain the rationale for sharing disease surveillance information - Describe the target audience of disease surveillance data - Demonstrate why periodic reports are a fundamental component of effective disease surveillance systems - Identify how ministries can communicate surveillance data and collaborate on investigations using a One Health approach |
| 1.12 | Surveillance  Monitoring | - Identify monitoring and evaluation in the context of overseeing a surveillance system - Explain indicator and target in the context of monitoring a surveillance system - Recognize key indicators to monitor surveillance timeliness and completeness at the district level - Explain the actions that can be taken to improve district-level disease surveillance |
|  | Case Study: Avian Influenza Surveillance in Country Z | - List and describe the steps of the surveillance cycle - Describe the purpose and use of local surveillance data - Complete a surveillance case report form - Calculate and interpret incidence rates and case-fatality rates - Identify and correct data errors - Summarize and interpret surveillance data by person, place, and time using rates, tables, and charts - Identify how One Health can be applied to strengthening surveillance systems and how different sectors can contribute to surveillance |
| 2.01 | Outbreak Investigation: Recognizing an Outbreak | - Determine when to investigate an outbreak - Develop clear investigation objectives |
| 2.02a | Outbreak Investigation: Initiating an Outbreak Investigation | - Identify the members needed for your outbreak investigation team - Confirm the existence of an outbreak - Construct an outbreak case definition - Find cases systematically - Develop an analysis plan |
| 2.02b | Outbreak Investigation: Descriptive Phase | - Summarize cases based on Time, Place and Person |
| 2.03a | Outbreak Investigation: Analysis | - Develop a hypothesis - Discuss ways to evaluate that hypothesis - Describe different modes of transmission of communicable diseases |
| 2.03b | Outbreak Investigation: Response | - Discuss strategies for outbreak control - Apply a One Health approach to an outbreak investigation and response |
| 2.04 | Linking with Laboratory | - Describe the interaction that should occur between epidemiology and laboratory staff   - on an ongoing basis   - when an outbreak investigation begins   - during the outbreak investigation   - after an outbreak investigation - Understand the common reasons for sample rejection |
| 2.05 | Biosafety and Biosecurity | - Define Biosafety and Biosecurity   - Laboratory setting   - Field Setting - Understand measures to be taken for Biosafety and Biosecurity   - Laboratory setting   - Field Setting |
| 2.06 | Risk Communication and Community Engagement | - Describe various communication types (as part of health response) - Explain risk communication, misinformation, disinformation and community engagement - Understand the fundamentals of effective risk communication - Understand the fundamentals of community engagement - Use the basic participatory epidemiology methods - Apply the multisectoral, One Health approach to risk communication and community engagement activities |
| 2.07 | Oral Presentation:  Plan, Prepare, Deliver | - Describe the characteristics of a strong presentation - List the steps of the presentation strategy - Develop a SOCO - Describe and organize a presentation using the traditional structure of a technical presentation - Deliver a brief presentation to an audience |
|  | Case Study: Avian Influenza Outbreak Investigation | - Conduct joint outbreak investigation in a scientific manner - Understand the importance of timely information sharing during outbreaks - Outline the principles of containment at source - Implement appropriate control measures to contain H5N1 poultry Outbreaks - Describe the roles and responsibilities of stakeholders in a multisectoral rapid response team - Design a press release to be used during an outbreak - Conclude the importance of a multidisciplinary approach in controlling and containing zoonotic disease outbreaks. |
| 3.01 | Joint Risk Assessment (JRA) for Frontline | - Outline the JRA process - Discuss the fundamentals of risk assessment - Create the risk pathway associated with zoonotic diseases - Develop questions for risk assessment - Explain the process for characterizing the risk |
| 3.02 | Water and Sanitation, Hygiene | - Describe WASH-relevant public health problems - Describe water safety and quality in promoting One Health - Explain sanitation and wastewater related issues - Monitoring of WASH services |
| 3.03 | Vaccination | - Describe the different strategies of vaccination for disease control and prevention - Evaluate different vaccination approaches - Describe the vaccination schedule for animal diseases in India - List of vaccination programs implemented for the control of the disease |
| 3.04 | Problem Analysis | - Analyse a single sector or a multisectoral public health problem systematically - Identify and organize root causes of a problem by using a cause-and-effect diagram - Differentiate causes between those you do and do not have control over - Develop an improvement plan for an appropriate key cause |
| 3.05a | International Health Regulations-Performance of Veterinary Services (IHR-PVS) | - Understand the scope of IHR- Performance Veterinary Services (PVS) - Describe the purpose and tools of Performance Veterinary Services - Understand inter-sector collaboration between IHR (WHO) and PVS(WOAH) Pathway |
|  | Animal Quarantine Certification Services | - To explain the ingress of any Exotic Livestock Diseases into India through importation of livestock & livestock products as per the provisions of Livestock Importation Act as amended by the Livestock Importation (Amendment) Act, 2001 (5.7.2001) - Regulations orders and SPS standards of the country issued - To provide an internationally accepted certification service - To export and increase international trade of livestock and livestock products - To inspect and register the plants/mills exporting the animal by-products |
| 3.06 | Stakeholder Analysis and Engagement | - Describe the terms – “Stakeholder” and “Stakeholder Analysis” - Understand why perform stakeholder analysis for one health issues - List the steps of stakeholder analysis - Identify, map and categorise stakeholders for one health issues |
| 3.07 | Hands on QGIS | - Prepare spot map for outbreak data - Prepare district wise-and block-wise area map |

**Supplementary Table 2. Coverage of COHFE frontline competencies by domain in the Field Epidemiology Programme in One Health (FEP OH) curriculum in India**

| **Domain** | **Domain Name** | **Total** | **Covered** | **Partially Covered** | **Not Covered** | **Total Coverage (%)** |
| --- | --- | --- | --- | --- | --- | --- |
| 1 | Foundational knowledge and skills | 26 | 11 | 5 | 10 | 61.5 |
| 2 | Surveillance systems | 30 | 17 | 8 | 5 | 83.3 |
| 3 | Field investigations | 37 | 21 | 11 | 5 | 86.5 |
| 4 | Disease management | 45 | 4 | 15 | 26 | 42.2 |
| 5 | Laboratory capacity | 27 | 14 | 6 | 7 | 74.1 |
| 6 | Infection prevention and control biosafety and biosecurity | 36 | 12 | 9 | 15 | 58.3 |
| 7 | Preparedness and response | 34 | 11 | 13 | 10 | 70.6 |
| 8 | Epidemiologic studies | 33 | 20 | 6 | 7 | 78.8 |
| 9 | Data management, biostatistics and informatics | 22 | 19 | 2 | 1 | 95.5 |
| 10 | Ecosystem health | 42 | 3 | 16 | 23 | 45.2 |
| 11 | Leadership and management | 39 | 11 | 15 | 13 | 66.7 |
| 12 | Communication and community engagement | 17 | 13 | 2 | 2 | 88.2 |
| 13 | Training | 25 | 1 | 4 | 20 | 20.0 |
| 14 | Ethics | 35 | 0 | 6 | 29 | 17.1 |
